# 2,2,6,6-Tetra­methyl-4-oxopiperidin-1-ium 4-chloro-3-nitro­benzoate

**DOI:** 10.1107/S1600536811025074

**Published:** 2011-07-06

**Authors:** Bohari M. Yamin, Norsakina Z. Zulkifli

**Affiliations:** a School of Chemical Sciences and Food Technology, Universiti Kebangsaan Malaysia, UKM 43500 Bangi Selangor, Malaysia

## Abstract

The title salt, C_9_H_18_NO^+^·C_7_H_3_ClNO_4_^−^, was obtained as an unexpected product of the reaction of 4-chloro-3-nitro­benzoyl isothio­cyanate with pyrrolidine. The six-membered ring of the 4-oxopiperidinium cation adopts a chair conformation. In the crystal structure, two cations and three anions are linked together by inter­molecular N—H⋯O and C—H⋯O hydrogen bonds and arranged diagonally along the *ac* face.

## Related literature

For related structures, see: Wang *et al.* (2008 ▶); Jasinski *et al.* (2009 ▶), Smith & Wermuth (2011 ▶). For bond-length data, see Allen *et al.* (1987 ▶). For puckering parameters, see: Cremer & Pople (1975 ▶). 
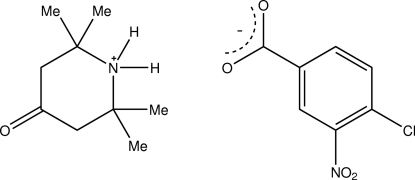


## Experimental

### 

#### Crystal data

C_9_H_18_NO^+^·C_7_H_3_ClNO_4_^−^
*M* *_r_* = 356.80
Triclinic, 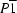

*a* = 7.9974 (10) Å
*b* = 10.3267 (13) Å
*c* = 11.9196 (15) Å
α = 109.101 (3)°
β = 96.785 (3)°
γ = 104.720 (3)°
*V* = 877.58 (19) Å^3^
*Z* = 2
Mo *K*α radiation
μ = 0.25 mm^−1^
*T* = 298 K
0.40 × 0.14 × 0.09 mm

#### Data collection

Bruker SMART APEX CCD area-detector diffractometer
Absorption correction: multi-scan (*SADABS*; Bruker, 2000 ▶) *T*_min_ = 0.908, *T*_max_ = 0.978
10082 measured reflections
3431 independent reflections
2268 reflections with *I* > 2σ(*I*)
*R*_int_ = 0.030

#### Refinement

*R*[*F* ^2^ > 2σ(*F* ^2^)] = 0.047
*wR*(*F* ^2^) = 0.126
*S* = 1.01
3431 reflections
229 parameters
2 restraints
H atoms treated by a mixture of independent and constrained refinement
Δρ_max_ = 0.25 e Å^−3^
Δρ_min_ = −0.18 e Å^−3^

### 

Data collection: *SMART* (Bruker, 2000 ▶); cell refinement: *SAINT* (Bruker, 2000 ▶); data reduction: *SAINT*; program(s) used to solve structure: *SHELXTL* (Sheldrick, 2008 ▶); program(s) used to refine structure: *SHELXTL*; molecular graphics: *SHELXTL*; software used to prepare material for publication: *SHELXTL*, *PARST* (Nardelli, 1995 ▶) and *PLATON* (Spek, 2009 ▶).

## Supplementary Material

Crystal structure: contains datablock(s) global, I. DOI: 10.1107/S1600536811025074/ff2016sup1.cif

Structure factors: contains datablock(s) I. DOI: 10.1107/S1600536811025074/ff2016Isup2.hkl

Supplementary material file. DOI: 10.1107/S1600536811025074/ff2016Isup3.cml

Additional supplementary materials:  crystallographic information; 3D view; checkCIF report

## Figures and Tables

**Table 1:** Hydrogen-bond geometry (Å, °)

*D*—H⋯*A*	*D*—H	H⋯*A*	*D*⋯*A*	*D*—H⋯*A*
N1—H1*A*⋯O3^i^	0.87 (2)	1.89 (2)	2.750 (2)	165
N1—H1*B*⋯O2^ii^	0.89 (1)	1.77 (1)	2.653 (2)	171
C3—H3*A*⋯O4^iii^	0.97	2.54	3.269 (3)	132
C8—H8*B*⋯O3^i^	0.96	2.54	3.297 (3)	136

Symmetry codes: (i) 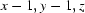; (ii) 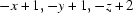; (iii) 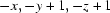.

**Fig. 1.**
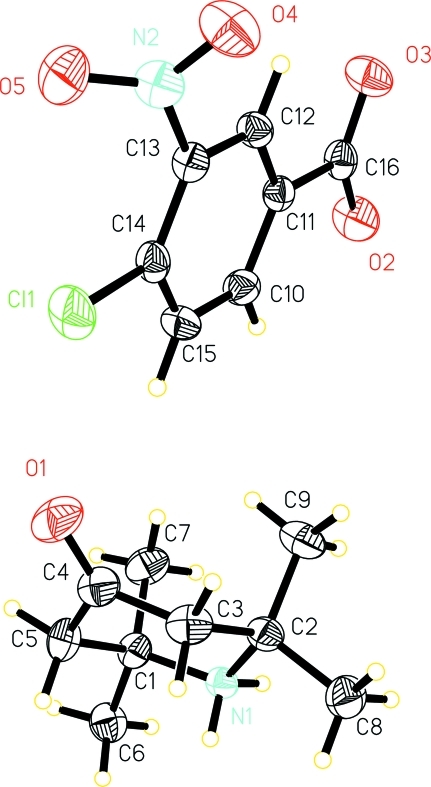
The molecular structure of (I), with displacement ellipsods drawn at the 50% probability level.

**Fig. 2.**
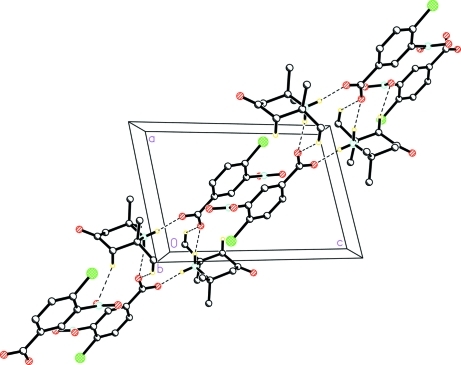
A packing diagram of (I) viewed down the b axis. Hydrogen bonds are shown by dashed lines.

## supplementary crystallographic information

### Comment

The title salt is an unexpected product of the reaction of 4-chloro-3- nitro-benzoylisothiocyanate with pyrrolidine. The expected product was *N*-(4-chloro-3-nitrobenzoyl)-*N*'-(pyrrolidin-1-yl)thiourea. The salt consists of 2,2,6,6-tetramethylpiperidinium-4-one cation and 4-chloro-3-nitrobenzoate anion (Fig.1) indicating the opening of pyrrolidine ring and involvement of acetone solvent in the reaction mechanism. The piperidinium ring adopts a chair conformation with puckering parameters (Cremer & Pople, 1975) Q, θ and φ, of 0.507 (2) Å, 3.4 (3)° and 207 (6)°, respectively. The bond lengths and angles are in normal range (Allen *et al.*, 1987) and comparable to those in piperidinium 3-hydroxy-2-naphthoate (Wang *et al.*, 2008) and 4-carbamoylpiperidinium 5-nitrosalicylate (Smith & Wermuth, 2011). All atoms of the benzoate anion are essentially coplanar with the benzene ring except O4 and O5, which are deviated from the plane by 0.690 (2) and 0.880 (2) Å, respectively. In the crystal structure, two cations and three anions are linked together by intermolecular hydrogen bonds (symmetry codes as in Table 2) and arranged diagonally along the ac face (Fig.2).

### Experimental

A solution of 4-chloro-3-nitrobenzoylisothiocyanate (2.42 g, 0.01 mol) in 30 ml acetone was added into a flask containing 30 ml acetone solution of pyyrolidine (0.71 g, 0.01 mol).The mixture was refluxed for 1 h. Then, the solution was filtered-off and left to evaporate at room temperature. The colourless solid was obtained after one day of evaporation (yield 83%, m.p 473.1–474.3 K).

### Refinement

H atoms on the parent carbon atoms were positioned geometrically with C—H= 0.96–0.98 Å and constrained to ride on their parent atoms with *U*_iso_(H)= *xU*_eq_(parent atom) where *x*=1.5 for CH_3_ group and 1.2 for CH_2_ and CH groups.

### Crystal data

**Table:**

C_9_H_18_NO^+^·C_7_H_3_ClNO_4_^−^	*Z* = 2
*M**_r_* = 356.80	*F*(000) = 376
Triclinic, *P*1	*D*_x_ = 1.350 Mg m^−^^3^
Hall symbol: -P 1	Melting point = 447.3–448.1 K
*a* = 7.9974 (10) Å	Mo *K*α radiation, λ = 0.71073 Å
*b* = 10.3267 (13) Å	Cell parameters from 1985 reflections
*c* = 11.9196 (15) Å	θ = 1.8–26.0°
α = 109.101 (3)°	µ = 0.25 mm^−^^1^
β = 96.785 (3)°	*T* = 298 K
γ = 104.720 (3)°	Slab, colourless
*V* = 877.58 (19) Å^3^	0.40 × 0.14 × 0.09 mm

### Data collection

**Table:**

Bruker SMART APEX CCD area-detector diffractometer	3431 independent reflections
Radiation source: fine-focus sealed tube	2268 reflections with *I* > 2σ(*I*)
graphite	*R*_int_ = 0.030
Detector resolution: 83.66 pixels mm^-1^	θ_max_ = 26.0°, θ_min_ = 1.8°
ω scans	*h* = −9→9
Absorption correction: multi-scan (*SADABS*; Bruker, 2000)	*k* = −12→12
*T*_min_ = 0.908, *T*_max_ = 0.978	*l* = −14→14
10082 measured reflections	

### Refinement

**Table:**

Refinement on *F*^2^	Primary atom site location: structure-invariant direct methods
Least-squares matrix: full	Secondary atom site location: difference Fourier map
*R*[*F*^2^ > 2σ(*F*^2^)] = 0.047	Hydrogen site location: inferred from neighbouring sites
*wR*(*F*^2^) = 0.126	H atoms treated by a mixture of independent and constrained refinement
*S* = 1.01	*w* = 1/[σ^2^(*F*_o_^2^) + (0.0655*P*)^2^ + 0.0942*P*] where *P* = (*F*_o_^2^ + 2*F*_c_^2^)/3
3431 reflections	(Δ/σ)_max_ < 0.001
229 parameters	Δρ_max_ = 0.25 e Å^−^^3^
2 restraints	Δρ_min_ = −0.18 e Å^−^^3^

### Special details

**Table:**

Geometry. All e.s.d.'s (except the e.s.d. in the dihedral angle between two l.s. planes) are estimated using the full covariance matrix. The cell e.s.d.'s are taken into account individually in the estimation of e.s.d.'s in distances, angles and torsion angles; correlations between e.s.d.'s in cell parameters are only used when they are defined by crystal symmetry. An approximate (isotropic) treatment of cell e.s.d.'s is used for estimating e.s.d.'s involving l.s. planes.
Refinement. Refinement of *F*^2^ against ALL reflections. The weighted *R*-factor *wR* and goodness of fit *S* are based on *F*^2^, conventional *R*-factors *R* are based on *F*, with *F* set to zero for negative *F*^2^. The threshold expression of *F*^2^ > σ(*F*^2^) is used only for calculating *R*-factors(gt) *etc*. and is not relevant to the choice of reflections for refinement. *R*-factors based on *F*^2^ are statistically about twice as large as those based on *F*, and *R*- factors based on ALL data will be even larger.

### Fractional atomic coordinates and isotropic or equivalent isotropic displacement parameters (Å^2^)

**Table:**

	*x*	*y*	*z*	*U*_iso_*/*U*_eq_	
Cl1	0.10661 (8)	0.44819 (7)	0.35541 (6)	0.0712 (2)	
O1	0.1974 (3)	0.1497 (2)	0.57035 (16)	0.0823 (6)	
O2	0.7048 (2)	0.76828 (18)	0.88016 (14)	0.0742 (5)	
O3	0.7880 (2)	0.93410 (17)	0.80065 (14)	0.0624 (5)	
O4	0.3749 (3)	0.8614 (2)	0.41307 (17)	0.0802 (6)	
O5	0.3627 (3)	0.6544 (2)	0.28559 (16)	0.0862 (6)	
N1	0.1289 (2)	0.11990 (18)	0.88732 (15)	0.0386 (4)	
H1A	0.0241 (16)	0.0568 (17)	0.8704 (17)	0.042 (6)*	
H1B	0.176 (3)	0.151 (2)	0.9662 (10)	0.052 (6)*	
N2	0.3733 (2)	0.7373 (2)	0.38805 (18)	0.0578 (5)	
C1	0.2358 (3)	0.0336 (2)	0.81718 (18)	0.0452 (5)	
C2	0.0998 (3)	0.2474 (2)	0.86054 (19)	0.0460 (5)	
C3	0.0313 (3)	0.1960 (3)	0.7231 (2)	0.0561 (6)	
H3A	−0.0898	0.1326	0.7014	0.067*	
H3B	0.0300	0.2788	0.7019	0.067*	
C4	0.1398 (3)	0.1179 (3)	0.6502 (2)	0.0559 (6)	
C5	0.1637 (3)	−0.0069 (3)	0.68090 (19)	0.0575 (6)	
H5A	0.2447	−0.0452	0.6356	0.069*	
H5B	0.0505	−0.0824	0.6552	0.069*	
C6	0.2039 (3)	−0.1004 (2)	0.8498 (2)	0.0587 (6)	
H6A	0.2490	−0.0723	0.9354	0.088*	
H6B	0.2636	−0.1627	0.8047	0.088*	
H6C	0.0791	−0.1504	0.8297	0.088*	
C7	0.4326 (3)	0.1167 (3)	0.8553 (2)	0.0642 (7)	
H7A	0.4688	0.1553	0.9425	0.096*	
H7B	0.4550	0.1943	0.8259	0.096*	
H7C	0.4984	0.0532	0.8216	0.096*	
C8	−0.0406 (3)	0.2880 (3)	0.9280 (2)	0.0639 (7)	
H8A	0.0056	0.3221	1.0141	0.096*	
H8B	−0.1435	0.2050	0.9045	0.096*	
H8C	−0.0723	0.3627	0.9080	0.096*	
C9	0.2697 (3)	0.3757 (2)	0.9055 (2)	0.0682 (7)	
H9A	0.3208	0.3943	0.9885	0.102*	
H9B	0.2425	0.4593	0.9006	0.102*	
H9C	0.3525	0.3541	0.8558	0.102*	
C10	0.4375 (3)	0.5931 (2)	0.67661 (19)	0.0457 (5)	
H10A	0.4547	0.5605	0.7398	0.055*	
C11	0.5459 (2)	0.7263 (2)	0.68617 (17)	0.0379 (5)	
C12	0.5203 (2)	0.7728 (2)	0.59146 (17)	0.0410 (5)	
H12A	0.5910	0.8622	0.5968	0.049*	
C13	0.3893 (3)	0.6860 (2)	0.48870 (18)	0.0419 (5)	
C14	0.2792 (3)	0.5541 (2)	0.47945 (18)	0.0442 (5)	
C15	0.3041 (3)	0.5083 (2)	0.5742 (2)	0.0501 (5)	
H15A	0.2311	0.4200	0.5696	0.060*	
C16	0.6921 (3)	0.8182 (2)	0.79869 (18)	0.0444 (5)	

### Atomic displacement parameters (Å^2^)

**Table:**

	*U*^11^	*U*^22^	*U*^33^	*U*^12^	*U*^13^	*U*^23^
Cl1	0.0475 (4)	0.0709 (4)	0.0599 (4)	0.0018 (3)	−0.0096 (3)	−0.0004 (3)
O1	0.0921 (14)	0.1105 (15)	0.0579 (11)	0.0264 (11)	0.0259 (10)	0.0492 (11)
O2	0.0933 (13)	0.0718 (11)	0.0368 (9)	0.0035 (10)	−0.0101 (8)	0.0193 (8)
O3	0.0499 (9)	0.0538 (10)	0.0625 (10)	−0.0038 (8)	−0.0101 (8)	0.0179 (8)
O4	0.0923 (14)	0.0673 (12)	0.0808 (13)	0.0234 (10)	−0.0083 (10)	0.0380 (10)
O5	0.1131 (16)	0.0961 (14)	0.0418 (10)	0.0258 (12)	0.0073 (10)	0.0245 (10)
N1	0.0376 (10)	0.0401 (10)	0.0317 (9)	0.0051 (8)	0.0009 (8)	0.0127 (8)
N2	0.0518 (12)	0.0640 (14)	0.0517 (13)	0.0125 (10)	−0.0058 (9)	0.0241 (11)
C1	0.0452 (12)	0.0525 (13)	0.0394 (11)	0.0177 (10)	0.0074 (9)	0.0178 (10)
C2	0.0479 (12)	0.0425 (12)	0.0479 (12)	0.0111 (9)	0.0079 (10)	0.0203 (10)
C3	0.0547 (14)	0.0666 (15)	0.0555 (14)	0.0179 (12)	0.0054 (11)	0.0369 (12)
C4	0.0526 (13)	0.0719 (16)	0.0384 (12)	0.0106 (12)	0.0014 (10)	0.0240 (11)
C5	0.0676 (16)	0.0656 (15)	0.0383 (12)	0.0252 (12)	0.0132 (11)	0.0142 (11)
C6	0.0660 (15)	0.0537 (14)	0.0607 (15)	0.0240 (12)	0.0115 (12)	0.0234 (12)
C7	0.0469 (14)	0.0842 (18)	0.0701 (16)	0.0235 (13)	0.0146 (12)	0.0365 (14)
C8	0.0691 (16)	0.0586 (15)	0.0727 (17)	0.0284 (13)	0.0236 (13)	0.0263 (13)
C9	0.0698 (17)	0.0487 (14)	0.0756 (18)	0.0016 (12)	0.0062 (13)	0.0262 (13)
C10	0.0469 (12)	0.0481 (12)	0.0415 (12)	0.0116 (10)	0.0106 (9)	0.0178 (10)
C11	0.0354 (10)	0.0399 (11)	0.0344 (10)	0.0119 (9)	0.0068 (8)	0.0092 (9)
C12	0.0361 (11)	0.0389 (11)	0.0426 (12)	0.0084 (9)	0.0056 (9)	0.0120 (9)
C13	0.0387 (11)	0.0469 (12)	0.0385 (11)	0.0148 (9)	0.0058 (9)	0.0136 (9)
C14	0.0341 (11)	0.0445 (12)	0.0416 (12)	0.0106 (9)	0.0043 (9)	0.0030 (9)
C15	0.0436 (12)	0.0403 (12)	0.0575 (14)	0.0029 (9)	0.0120 (10)	0.0146 (10)
C16	0.0414 (12)	0.0472 (13)	0.0357 (11)	0.0133 (10)	0.0015 (9)	0.0068 (10)

### Geometric parameters (Å, °)

**Table:**

Cl1—C14	1.726 (2)	C6—H6A	0.9600
O1—C4	1.207 (3)	C6—H6B	0.9600
O2—C16	1.245 (3)	C6—H6C	0.9600
O3—C16	1.238 (3)	C7—H7A	0.9600
O4—N2	1.213 (2)	C7—H7B	0.9600
O5—N2	1.219 (2)	C7—H7C	0.9600
N1—C1	1.514 (3)	C8—H8A	0.9600
N1—C2	1.517 (3)	C8—H8B	0.9600
N1—H1A	0.874 (9)	C8—H8C	0.9600
N1—H1B	0.888 (9)	C9—H9A	0.9600
N2—C13	1.467 (3)	C9—H9B	0.9600
C1—C7	1.520 (3)	C9—H9C	0.9600
C1—C6	1.524 (3)	C10—C15	1.383 (3)
C1—C5	1.536 (3)	C10—C11	1.387 (3)
C2—C8	1.521 (3)	C10—H10A	0.9300
C2—C3	1.529 (3)	C11—C12	1.377 (3)
C2—C9	1.529 (3)	C11—C16	1.516 (3)
C3—C4	1.494 (3)	C12—C13	1.379 (3)
C3—H3A	0.9700	C12—H12A	0.9300
C3—H3B	0.9700	C13—C14	1.383 (3)
C4—C5	1.499 (3)	C14—C15	1.372 (3)
C5—H5A	0.9700	C15—H15A	0.9300
C5—H5B	0.9700		
			
C1—N1—C2	120.56 (16)	H6B—C6—H6C	109.5
C1—N1—H1A	103.8 (13)	C1—C7—H7A	109.5
C2—N1—H1A	106.4 (13)	C1—C7—H7B	109.5
C1—N1—H1B	108.5 (14)	H7A—C7—H7B	109.5
C2—N1—H1B	107.7 (13)	C1—C7—H7C	109.5
H1A—N1—H1B	109.6 (19)	H7A—C7—H7C	109.5
O4—N2—O5	124.2 (2)	H7B—C7—H7C	109.5
O4—N2—C13	117.4 (2)	C2—C8—H8A	109.5
O5—N2—C13	118.4 (2)	C2—C8—H8B	109.5
N1—C1—C7	111.61 (17)	H8A—C8—H8B	109.5
N1—C1—C6	105.17 (17)	C2—C8—H8C	109.5
C7—C1—C6	109.29 (17)	H8A—C8—H8C	109.5
N1—C1—C5	107.97 (16)	H8B—C8—H8C	109.5
C7—C1—C5	111.59 (19)	C2—C9—H9A	109.5
C6—C1—C5	111.04 (18)	C2—C9—H9B	109.5
N1—C2—C8	105.96 (17)	H9A—C9—H9B	109.5
N1—C2—C3	107.28 (17)	C2—C9—H9C	109.5
C8—C2—C3	110.62 (19)	H9A—C9—H9C	109.5
N1—C2—C9	111.61 (17)	H9B—C9—H9C	109.5
C8—C2—C9	109.71 (19)	C15—C10—C11	120.8 (2)
C3—C2—C9	111.51 (19)	C15—C10—H10A	119.6
C4—C3—C2	113.43 (18)	C11—C10—H10A	119.6
C4—C3—H3A	108.9	C12—C11—C10	119.08 (18)
C2—C3—H3A	108.9	C12—C11—C16	120.59 (18)
C4—C3—H3B	108.9	C10—C11—C16	120.33 (18)
C2—C3—H3B	108.9	C11—C12—C13	119.65 (19)
H3A—C3—H3B	107.7	C11—C12—H12A	120.2
O1—C4—C3	122.5 (2)	C13—C12—H12A	120.2
O1—C4—C5	123.1 (2)	C12—C13—C14	121.47 (19)
C3—C4—C5	114.4 (2)	C12—C13—N2	117.49 (18)
C4—C5—C1	113.22 (18)	C14—C13—N2	121.03 (18)
C4—C5—H5A	108.9	C15—C14—C13	118.79 (19)
C1—C5—H5A	108.9	C15—C14—Cl1	118.87 (17)
C4—C5—H5B	108.9	C13—C14—Cl1	122.29 (17)
C1—C5—H5B	108.9	C14—C15—C10	120.2 (2)
H5A—C5—H5B	107.7	C14—C15—H15A	119.9
C1—C6—H6A	109.5	C10—C15—H15A	119.9
C1—C6—H6B	109.5	O3—C16—O2	126.28 (19)
H6A—C6—H6B	109.5	O3—C16—C11	117.47 (19)
C1—C6—H6C	109.5	O2—C16—C11	116.24 (19)
H6A—C6—H6C	109.5		
			
C2—N1—C1—C7	73.0 (2)	C16—C11—C12—C13	178.44 (17)
C2—N1—C1—C6	−168.64 (17)	C11—C12—C13—C14	1.6 (3)
C2—N1—C1—C5	−50.0 (2)	C11—C12—C13—N2	−177.04 (18)
C1—N1—C2—C8	168.99 (17)	O4—N2—C13—C12	−47.1 (3)
C1—N1—C2—C3	50.8 (2)	O5—N2—C13—C12	131.6 (2)
C1—N1—C2—C9	−71.6 (2)	O4—N2—C13—C14	134.3 (2)
N1—C2—C3—C4	−49.4 (2)	O5—N2—C13—C14	−47.1 (3)
C8—C2—C3—C4	−164.50 (19)	C12—C13—C14—C15	−1.2 (3)
C9—C2—C3—C4	73.1 (3)	N2—C13—C14—C15	177.42 (19)
C2—C3—C4—O1	−128.3 (2)	C12—C13—C14—Cl1	176.34 (15)
C2—C3—C4—C5	54.4 (3)	N2—C13—C14—Cl1	−5.0 (3)
O1—C4—C5—C1	129.6 (2)	C13—C14—C15—C10	−0.2 (3)
C3—C4—C5—C1	−53.1 (3)	Cl1—C14—C15—C10	−177.79 (16)
N1—C1—C5—C4	47.4 (2)	C11—C10—C15—C14	1.1 (3)
C7—C1—C5—C4	−75.6 (2)	C12—C11—C16—O3	−1.2 (3)
C6—C1—C5—C4	162.25 (19)	C10—C11—C16—O3	177.94 (19)
C15—C10—C11—C12	−0.7 (3)	C12—C11—C16—O2	179.46 (19)
C15—C10—C11—C16	−179.79 (18)	C10—C11—C16—O2	−1.4 (3)
C10—C11—C12—C13	−0.7 (3)		

### Hydrogen-bond geometry (Å, °)

**Table:**

*D*—H···*A*	*D*—H	H···*A*	*D*···*A*	*D*—H···*A*
N1—H1A···O3^i^	0.87 (2)	1.89 (2)	2.750 (2)	165
N1—H1B···O2^ii^	0.89 (1)	1.77 (1)	2.653 (2)	171
C3—H3A···O4^iii^	0.97	2.54	3.269 (3)	132
C8—H8B···O3^i^	0.96	2.54	3.297 (3)	136

Symmetry codes: (i) *x*−1, *y*−1, *z*; (ii) −*x*+1, −*y*+1, −*z*+2; (iii) −*x*, −*y*+1, −*z*+1.

## References

[bb1] Allen, F. H., Kennard, O., Watson, D. G., Brammer, L., Orpen, A. G. & Taylor, R. (1987). *J. Chem. Soc. Perkin Trans. 2*, pp. S1–19.

[bb2] Bruker (2000). *SADABS*, *SMART* and *SAINT* Bruker AXS Inc., Madison, Wisconsin, USA.

[bb3] Cremer, D. & Pople, J. A. (1975). *J. Am. Chem. Soc.* **97**, 1354–1358.

[bb4] Jasinski, J. P., Butcher, R. J., Yathirajan, H. S., Mallesha, L. & Mohana, K. N. (2009). *Acta Cryst.* E**65**, o2365–o2366. 10.1107/S1600536809035363 PMC2970333 21577832

[bb5] Nardelli, M. (1995). *J. Appl. Cryst.* **28**, 659.

[bb6] Sheldrick, G. M. (2008). *Acta Cryst.* A**64**, 112–122. 10.1107/S0108767307043930 18156677

[bb7] Smith, G. & Wermuth, U. D. (2011). *Acta Cryst.* E**67**, o122. 10.1107/S1600536810050129 PMC3050397 21522633

[bb8] Spek, A. L. (2009). *Acta Cryst.* D**65**, 148–155. 10.1107/S090744490804362X PMC2631630 19171970

[bb9] Wang, Y.-T., Tang, G.-M., Zhang, Y.-C. & Wan, W.-Z. (2008). *Acta Cryst.* E**64**, o1753. 10.1107/S1600536808025567 PMC2960605 21201735

